# Patient Satisfaction and Supportive Care Pathways in a German Head and Neck Tumor Center: A Prospective Cross-Sectional Study

**DOI:** 10.3390/healthcare14091192

**Published:** 2026-04-29

**Authors:** Mario Scheurer, Philip Haller, Johannes Schulze, Stefan Kist, Robin Kasper, Lukas Greber, Alisa Schramm, Majeed Rana, Alexander Schramm, Stefan Repky, Andreas Sakkas, Marcel Ebeling, Frank Wilde

**Affiliations:** 1 Department of Oral and Plastic Maxillofacial Surgery, Military Hospital Ulm, 89081 Ulm, Germany; 2 Department of Oral and Maxillofacial Surgery, University Hospital Ulm, 89081 Ulm, Germany; 3 Medical Faculty, University Ulm, 89081 Ulm, Germany; 4 Department of Otorhinolaryngology, Military Hospital Ulm, 89081 Ulm, Germany; 5 Department of Oral and Plastic Maxillofacial Surgery, Medical University Vienna, 1090 Vienna, Austria; 6 Institute of Epidemiology and Medical Biometry, University Ulm, 89075 Ulm, Germany

**Keywords:** patient-reported outcomes measures, interdisciplinary oncology care, supportive care services, discharge management, health-related quality of life, oncology care pathways, patient-centered care, hospital quality management, quality of care

## Abstract

**Background/Objectives:** Patient satisfaction and supportive care are key quality indicators in certified Head and Neck Cancer Centers (HNCC). We assessed patient-reported experiences across diagnostic staging and surgical treatment pathways, focusing on discharge management and supportive service integration. **Materials and Methods:** In this prospective cross-sectional study, 84 inpatients were surveyed at the time of hospital discharge after diagnostic tumor staging (*n* = 45) or surgical treatment (*n* = 39) at a German tertiary HNCC. Phase-specific standardized questionnaires with five-point Likert scales were analyzed using Pearson’s chi-square and Fisher’s exact tests. Associations of sex and treatment intensity with satisfaction and supportive care utilization were explored descriptively and in an exploratory manner. **Results:** Overall ratings were high across both cohorts for admission processes, inpatient organization and medical and nursing care, with no statistically significant between-group differences (*p* > 0.05). Information regarding diagnostic and perioperative procedures was rated very positively in both groups. Discharge-related items were generally favorable. However, patients who underwent surgery reported greater uncertainty and lower reported utilization of formal discharge management. This difference did not reach statistical significance (*p* = 0.0559) and should therefore be interpreted as a non-significant trend toward less positive evaluation compared with diagnostic patients. Supportive services were rated predominantly good to very good by users (>95% positive ratings). Utilization differed by treatment intensity: Speech therapy was more frequent in operative patients (*p* < 0.001) and social work counseling was offered and utilized more often in patients undergoing extensive surgery (*p* = 0.042 and *p* = 0.027, respectively). Overall dissatisfaction was strongly associated with perceived deficiencies in information on diagnostic procedures and tumor-related counseling (both *p* < 0.001), whereas waiting time for surgery was not associated with negative overall ratings. **Conclusions:** Patient satisfaction was consistently high across diagnostic and surgical pathways. Adequate, transparent and repeated information, particularly on diagnostics and tumor counseling, was strongly associated with higher overall satisfaction, whereas objective timing metrics were not associated with negative ratings. Discharge management may represent a sensitive transition point, particularly after extensive surgery and may therefore be a relevant target for further optimization and proactive integration of supportive care services. Sex-specific findings were limited and should be interpreted cautiously due to small subgroup sizes.

## 1. Introduction

Head and neck cancer (HNC) represents a major global healthcare burden. Within this heterogenous group of malignancies, head and neck squamous cell carcinoma (HNSCC) constitutes the predominant histological subtype, accounting for approximately 950,000 newly diagnosed cases each year and ranking among the most common malignant tumors worldwide [1,2,3]. Despite advances in multidisciplinary treatment strategies, HNC remains associated with substantial mortality, accounting for more than 480,000 disease-related deaths annually. Epidemiological data demonstrate a continued rise in incidence, with projections indicating that annual new cases may exceed one million by 2025. By 2050, global incidence and mortality are expected to increase markedly, reaching over 1.5 million new cases and 823,000 deaths. Under scenarios assuming a sustained annual increase of approximately 3%, the global burden could escalate even further, exceeding 3.5 million new cases and 1.8 million deaths worldwide [1]. Five-year overall survival rates vary depending on tumor stage and risk profile, ranging from approximately 40% to 80% [1,4,5]. Against this epidemiological background, the management of HNC patients represents a major current and future challenge for healthcare systems [2]. High-quality care requires individualized, patient-specific treatment strategies embedded within well-coordinated multimodal concepts, balancing optimal oncologic outcomes with functional preservation and quality of life, particularly in the setting of increasingly constrained healthcare resources and capacities [6,7,8]. In oncologic care, patient satisfaction, health-related quality of life (HRQoL) and individual lifestyle goals can serve as surrogate markers of treatment quality and may additionally influence overall outcomes within a holistic, patient-centered framework [9,10,11,12].

HNC management, involving surgery, plastic reconstruction, radiotherapy, systemic therapy and rehabilitation, requires close interdisciplinary collaboration [2,7,13,14]. Despite growing emphasis on patient-centered therapy strategies and outcomes, few studies have systematically assessed satisfaction with supportive care, discharge management and aftercare in a German Head and Neck Cancer Center (HNCC) [15]. The systematic assessment of patient experience and satisfaction has become essential for evaluating healthcare quality, complementing traditional measures of process quality, clinical safety and therapeutic effectiveness [16,17]. Data demonstrate strong positive associations between patient experience and treatment adherence, clinical outcomes and safety, indicating that satisfied patients are more likely to engage actively in their own care and to adhere to treatment guidelines [18,19,20,21]. Within HNC, patient satisfaction assumes a pivotal role given the complexity of this disease and its multimodal, often invasive treatment pathways, the chronic course of this disease and the substantial emotional burden associated with HNC diagnosis and therapy [2,14]. Patient satisfaction in oncologic setting is determined by multiple interconnected factors, particularly the quality of interpersonal and professional competencies of the treating and nursing staff, the coordination across multidisciplinary teams and the perceived timeliness of diagnostic and therapeutic processes [22,23]. Qualitative oncology data reported by Mathews et al. suggest that patient satisfaction is driven by empathic clinician–patient engagement, effective communication and a shared understanding of the urgency and necessity of clinical intervention [22]. Prolonged waiting periods, whether before consultation, diagnosis, or treatment represent one of the most consistent sources of dissatisfaction in oncology [24]. These findings highlight that timeliness and communication constitute modifiable components of supportive care pathways. At the institutional level, structured patient satisfaction surveys have become integral elements of hospital quality management systems [25,26,27]. When implemented in a systematic and standardized manner, they may provide actionable insights into key domains such as communication processes, access to care, discharge planning and continuity of care, which are particularly relevant in HNCC [27]. Moreover, systematically collecting patient satisfaction data has been associated with continuous quality improvement and enhanced accountability, particularly through the identification of best-practice models across healthcare institutions [28]. In head and neck oncology, surgical treatment is frequently associated with significant functional, aesthetic, psychosocial impairment and treatment-associated morbidity that can substantially affect patients’ quality of life (QOL) [2,11,29]. Despite major advances in oncologic surgical and reconstructive techniques and therapies, the systematic assessment of patient-reported outcomes measures (PROM), patient satisfaction and symptoms, as well as their integration into perioperative, adjuvant and supportive care pathways remain limited and underexplored [30,31]. Multimodal HNC oncologic care requires close interdisciplinary coordination among surgery (including dental assessment), oncology, nursing (including specialized oncology nursing), rehabilitation, nutrition counseling, speech and swallowing therapy, social services counseling and psycho-oncological support, each individually and collectively influencing patient’s perceived QOL [32,33]. Consequently, a comprehensive understanding of patient experience within structured clinical and supportive care pathways is fundamental for establishing a veritable patient-centred surgical oncologic care. The present cross-sectional study was conducted to assess patient satisfaction and supportive care processes in a German HNCC embedded within a university-based comprehensive cancer center (CCC). By analyzing patient-reported experiences across the range of surgical oncology care, this study aims to identify specific determinants of patients’ satisfaction and to define areas for targeted improvement within institutional care pathways. Ultimately, these insights may contribute to the optimization of patient-centred outcomes and the establishment of evidence-based benchmarks for quality and satisfaction in surgical head and neck oncology.

## 2. Materials and Methods

### 2.1. Ethical Approval and Study Design

Ethical approval for this prospective cross-sectional study was obtained from the Ethics Committee of the University of Ulm, Germany (approval number: 162/22; approval date: 1 August 2022). The study was conducted in full accordance with the ethical standards of the institutional ethics committee and the Declaration of Helsinki and its later amendments [34,35].

The study was designed as an anonymous patient survey evaluating the quality of care at the HNCC of the Military and University Hospital Ulm, Department of Oral and Plastic Maxillofacial Surgery, a certified center in accordance with the guidelines of the German Cancer Society [Deutsche Krebsgesellschaft (DKG)] and OnkoZert (i.e., independent institute commissioned by the DKG to monitor the certification system for reviewing organ cancer centers and oncology centers in accordance with the relevant professional requirements). The study was designed as a prospective cross-sectional analysis, with patient-reported outcomes collected at a single predefined time point (hospital discharge). Data were collected prospectively using standardized, treatment-specific questionnaires developed for this purpose, aligned with the key quality indicators and process metrics defined in the DKG HNCC assessment framework. The questionnaire was institution-specific and was not formally validated regarding psychometric properties such as reliability and construct validity; however, it was developed under the guidance of an experienced auditor for DKG-certified HNCCs. Informed consent was obtained from all subjects involved in the study. In accordance with the ethics approval, all data were anonymized and no information allowing identification of individual patients was recorded. Between March 2023 and April 2025, primary cases of HNC treated at the Department of Oral and Plastic Maxillofacial Surgery, Military and University Hospital Ulm, were included. Reporting of this study followed the recommendations of the Strengthening the Reporting of Observational Studies in Epidemiology (STROBE) statement [36]. A completed STROBE checklist is provided as Supplementary Materials (Table S1).

### 2.2. Study Cohort

Patients were eligible for inclusion if they met the following criteria: (1) suspected or histologically confirmed HNC and (2) completion of a standardized questionnaire during either the diagnostic (i.e., staging) phase or the surgical treatment phase. Tumor entities were classified according to the standard International Statistical Classification of Diseases and Related Health Problems (10. Revision; ICD-10)-based classification of HNC. Eligible cases comprised malignant HNC defined as invasive neoplasms and in situ carcinomas of the upper aerodigestive tract, comprising ICD-10 codes C00–C14 (lip, oral cavity and pharynx), C30–C31 (nasal cavity and paranasal sinuses) and C32 (larynx), as well as corresponding in situ carcinomas (D00–D02) [37]. Tumors of the esophagus (C15) and salivary glands (C07–C08) were explicitly excluded.

Exclusion criteria were (1) absence of suspected or histologically confirmed HNC and (2) non-completion of the diagnostic (i.e., staging) or surgical evaluation questionnaire. For the analysis of patient-reported outcomes and supportive care processes, patients were assigned to either a diagnostic (staging) cohort (*n* = 45) or a surgical treatment cohort (*n* = 39). Ten incomplete questionnaires were excluded from the final analysis due to insufficiently completed items.

Due to the fully anonymous study design, no data on non-participants or individuals who declined participation were recorded. This should be considered when interpreting the results, as potential selection bias cannot be excluded.

### 2.3. Multidisciplinary Care Framework

Primary HNC cases were managed within a certified multidisciplinary HNCC. Care followed standardized institutional operating procedures and was coordinated across Oral and Maxillofacial Plastic Surgery, Otorhinolaryngology, Medical Oncology, Radiation Oncology, Radiology, Pathology, Nuclear medicine and supportive care services.

### 2.4. Protocol of Staging

All patients underwent a standardized primary diagnostic protocol, typically performed during inpatient admission. The staging process comprised a comprehensive clinical examination (including photographic documentation and intraoral scanning of the maxilla and mandible) by a board-certified oral and maxillofacial (OMF) surgent, cross-sectional imaging for tumor and nodal assessment using fluorine-18 fluorodeoxyglucose ([^18^F] FDG) positron emission tomography combined with computed tomography (PET/CT) or PET/magnetic resonance imaging (MRI) as indicated and high-resolution ultrasonography of the cervical region and the abdomen [38]. Histopathological confirmation was obtained by surgical sampling of clinically and radiologically suspicious lesions. Definitive diagnosis was established through microscopic and immunohistochemical analysis by a board-certified pathologist.

### 2.5. Interdisciplinary Head and Neck Tumor Board

Following completion of diagnostic staging, tumor classification was performed in accordance with the current TNM system [7]. All cases were subsequently formally registered and presented at the interdisciplinary head and neck tumor board (HNTB). The board convened on a weekly basis and comprised representatives from all core oncologic disciplines (Oral and Maxillofacial Plastic Surgery, Otorhinolaryngology, Medical Oncology, Radiation Oncology, Pathology, Radiology and Nuclear medicine).

During HNTB meetings, all available clinical, radiological and histopathological findings were comprehensively reviewed. Based on this interdisciplinary assessment, an individualized, guideline-concordant treatment strategy was determined by consensus [7]. Attention was paid to the extent of surgical resection and neck dissection, reconstructive strategies and indications for adjuvant therapies. In addition, patient-specific characteristics including comorbidities, functional status [e.g., Eastern Cooperative Oncology Group (ECOG) [39] and Karnofsky performance status (KPS) scale [40,41]] and individual treatment preferences were considered in the decision-making process. Each patient was discussed pre-therapeutically and post-therapeutically. HNTB recommendations were formally documented and communicated to the patient in a dedicated follow-up consultation.

### 2.6. Dental Assessment and Oral Focus Management

All patients, particularly those scheduled for radiotherapy or systemic oncologic therapy, underwent a mandatory structured dental assessment, including clinical and radiological evaluation, to identify and manage potential oral infectious foci. Dental sanitation and preventive measures (e.g., custom fluoride trays) were implemented to minimize treatment-related complications and to prevent delays in the initiation of oncologic therapy [7].

### 2.7. Supportive and Additional Health Care

Integrated supportive care was initiated at inpatient admission during staging investigations and, where applicable, reassessed upon admission for surgical treatment. Care was provided and documented in a standardized manner by the attending ward physician in close collaboration with nursing staff. This approach included specialized oncologic nursing care delivered through structured nursing consultations, encompassing systematic assessment and management of pain, nutritional status, wound care, airway and tracheostoma management, as well as psychosocial screening. Specialized oncology nurses coordinated supportive interventions, initiated referrals to allied health services (e.g., nutrition, speech and swallowing therapy, psycho-oncology, social services) and ensured continuity of care across inpatient treatment and discharge planning.

Nutritional counselling was provided based on systematic Nutritional Risk Screening (NRS 2002), as recommended by the European Society for Clinical Nutrition and Metabolism (ESPEN) [42]. The NRS 2002 evaluates nutritional status (including body mass index, recent weight loss and reduced dietary intake), disease severity-related metabolic stress and age-related risk factors. Patients identified as being at nutritional risk (NRS-2002 score ≥ 3) received structured, individualized nutritional counselling and, where indicated, further dietetic assessment and intervention aimed at preventing or treating cancer- or therapy-related malnutrition, reducing treatment-associated complications.

Speech and swallowing therapy were provided by certified speech-language pathologists for functional rehabilitation. Therapy was initiated on postoperative day (POD) 2–3 during the inpatient stay and delivered in a structured, stepwise manner based on standardized logopedic assessments [43,44]. Interventions focused on the evaluation and treatment of dysphagia and speech impairment, including clinical swallowing assessment, functional oral motor exercises, compensatory swallowing strategies and graded dietary recommendations. Dietary consistency and fluid texture were adjusted according to swallowing function, with documented progression from modified textures to regular oral intake as clinically appropriate. In patients with tracheostoma, therapy additionally comprises structured tracheal cannula management, including cuff deflation trials, speaking valve training, occlusion trials and assessment of readiness for decannulation. When these measures were successful, definitive surgical closure of the tracheostoma was performed under local anesthesia at the end of the inpatient stay. Therapeutic progress and recommendations were documented longitudinally and reassessed throughout hospitalization, with the aim of facilitating early functional recovery and restoring safe oral intake and effective communication.

Social work counselling was provided to address organizational, rehabilitative and socioeconomic needs, including counselling on disability-related benefits, coordination of applications for inpatient or outpatient rehabilitation, cooperation with statutory pension insurance providers and structured support for the transition from inpatient treatment to outpatient and home-care settings [45].

### 2.8. Psycho-Oncological Support

Psychological distress was routinely assessed using the National Comprehensive Cancer Network (NCCN) Distress Thermometer (DT). Patients were asked to indicate their level of distress by circling a value on a numeric DT ranging from 0 (no distress) to 10 (extreme distress). In addition, patients were requested to mark all applicable items across the predefined categories of physical, emotional, social, practical and spiritual or religious concerns. Free-text entry of additional concerns was possible in an open section. When indicated (DT score ≥ 5), patients were systematically referred to psycho-oncological counselling and, if required, to further therapeutic support [46]. Individual psycho-oncological recommendations were documented and incorporated into the individual care plan.

### 2.9. Documentation and Follow-Up

Comprehensive tumor documentation was performed in accordance with the mandatory documentation requirements of the DKG certification framework. All cases were systematically integrated into certified cancer registry structures and reported using standardized datasets defined within the DKG and OnkoZert quality assurance system. This ensured complete, guideline-concordant documentation of diagnostic, therapeutic and follow-up data across the entire treatment pathway.

Following completion of primary therapy, patients were enrolled in a structured follow-up program with a minimum duration of five years, comprising routine clinical examinations at three-month intervals during the first and second years and at six-month intervals from the third to the fifth year, complemented by scheduled imaging-based restaging once annually [7,38,47].

### 2.10. Questionnaire Design

For phase-specific assessment of patient-reported experiences, two standardized questionnaire versions were developed and administered according to the reason for hospital admission (diagnostic work-up/staging or surgical treatment). Questionnaire development was guided by treatment-specific quality indicators defined in the official data collection form for HNCC of the DKG, as elaborated by the Certification Commission for HNCC within the framework of certified oncology centers. The questionnaire was institution-specific and was not formally validated. The diagnostic questionnaire was applied to patients admitted for primary diagnostic evaluation or tumor staging, whereas the operative questionnaire was administered to patients undergoing surgical treatment for HNC. Both questionnaire versions were identical in structure and core content, differing only in items specifically addressing surgical tumor management. These surgery-related items included questions on the performance and type of tumor resection according to anatomical site, execution of neck dissection (ND), tracheostomy and reconstructive procedures with or without cutaneous or osseous free-flap transfer, as well as patients’ perceived adequacy of pre- and postoperative information regarding the surgical procedures and their functional consequences. This approach allowed for a comparative evaluation of patient experiences during the diagnostic and surgical phases of care. The structured questionnaire comprised nine thematic domains: admission processes, inpatient stay, surgical procedures, discharge management, psycho-oncological care, social services, nutritional counselling, speech and swallowing therapy and overall satisfaction. Additionally, treatment period and sex were documented. Responses were assessed using nominal or ordinal scales. Ordinal items were rated on a five-point Likert scale (“very good”, “good”, “fair”, “poor”, “not applicable”) to capture patients’ subjective perceptions and experiences [47]. In addition, open-ended free-text fields were provided to allow individualized responses, including feedback on aspects perceived as positive or in need of improvement. Completion of individual items was optional, as certain questions were only applicable to participants who had utilized specific services, diagnostic procedures or therapeutic interventions.

### 2.11. Data Collection

Data were collected using above-mentioned standardized and anonymized questionnaires. All applicable data protection regulations were strictly adhered to and no data enabling the identification of individual patients were collected. Questionnaires were completed at the end of the inpatient stay and anonymized in their entirety prior to analysis. Completed questionnaires were returned by patients into a sealed collection box located at the nursing station. The collection box was opened once annually in the presence of the principal investigator solely to document the number of returned questionnaires and to monitor study progress. The study was continued until it could be assumed that, after exclusion of all non-evaluable questionnaires, the predefined minimum sample size of 80 participants had been achieved. Only questionnaires meeting predefined case-wise validity criteria were included in the final analysis. No fixed a priori exclusion threshold based on the proportion of missing data was applied. Instead, questionnaires were excluded only when the pattern and extent of missingness rendered meaningful statistical evaluation impossible, such as omission of entire pages or questionnaire domains. Questionnaires with partial item non-response were retained and analyzed under the assumption of missing-at-random (MAR), with missing values treated accordingly in the statistical analysis. A questionnaire was considered valid if it permitted unambiguous assignment to the respective study group, contained no internally inconsistent or implausible responses, and met the minimum completeness required for case-wise inclusion.

### 2.12. Statistical Analysis

Statistical analyses were performed using SPSS software (version 26; IBM Corporation, Armonk, NY, USA). All statistical analyses were explicitly conducted as exploratory and data-driven. Given the ordinal nature of the data, non-parametric statistical tests were applied. For all questionnaire items, either Pearson’s chi-square test or Fisher’s exact test was used, depending on cell counts in the corresponding contingency tables. Resulting *p*-values are reported as descriptive indicators to assess approximate statistical associations and should not be interpreted as confirmatory evidence. No correction for multiple testing was applied due to the exploratory nature of the study. Subgroup analyses, including sex-stratified comparisons, were exploratory and not based on an a priori power calculation. These analyses are therefore statistically underpowered and must be interpreted with appropriate caution. For all tables without specific annotations, either the *p*-value derived from the Pearson’s chi-square test was considered valid or the exact Fisher test had already been applied and reported. Statistical significance was defined as *p* ≤ 0.05 and was indicated accordingly (* *p* ≤ 0.05, ** *p* ≤ 0.01, *** *p* ≤ 0.001). However, these thresholds must be interpreted within the exploratory framework of the analysis, and the reported *p*-values should be regarded as descriptive indicators rather than confirmatory evidence.

## 3. Results

### 3.1. Cohort Characteristics

The study cohort comprised a total of 84 cases, following exclusion of nine incompletely filled questionnaires. Of these, 45 cases (53.6%) were assigned to the diagnostic group and 39 cases (46.4%) to the operative group (Table 1).

#### 3.1.1. Diagnostic Group (Group 1)

Within this cohort 27 patients (60%) were males and 15 (33.3%) females. For 3 patients (6.7%), no information on sex was available. Regarding educational attainment, 7 patients (15.6%) reported high-school diploma and 3 patients (6.7%) indicated an advanced technical certificate. The largest proportions reported middle school degree (*n* = 14; 31.1%) or main school degree (*n* = 13; 28.9%). Eight patients (17.8%) chose not to disclose their educational level.

#### 3.1.2. Operative Group (Group 2)

In this cohort 19 patients (48.7%) were females and 18 (46.2%) were males. Sex was not specified in two cases (5.1%). With respect to educational attainment 7 patients (17.9%) reported having completion of high-school diploma and 2 (5.1%) advanced technical certificates. A middle school degree was reported by 10 patients (25.6%) and main school degree by 11 patients (28.2%). 2 patients (5.1%) indicated having no school qualification, while 7 patients (17.9%) declined to provide information.

### 3.2. Admission

Overall satisfaction with hospital admission and inpatient care was high across both cohorts. In the diagnostic group, 97.8% of respondents rated the admission process as very good or good, compared with 94.8% in the operative group. Negative ratings were rare in both cohorts, occurring in 2.2% of diagnostic and 5.2% of operative cases.

Regarding time interval between initial medical presentation and subsequent hospital admission (HNCC), most patients in both groups (group 1: 79.5; group 2: 64.9%) reported a waiting time of 1 to 3 weeks. Longer waiting periods more than 4 weeks were reported by 18.2% of patients in the diagnostic group and 27% in the operative group.

### 3.3. Inpatient Stay

Both cohorts provided highly favorable evaluations of inpatient care across all assessed domains. The organization of inpatient treatment was evaluated as very good or good by 93.3% of patients in the diagnostic group and 97.4% in the operative group.

The care provided by medical and nursing staff received particularly high ratings. Medical staff care was rated as very good or good by 97.8% (group 1) and 97.4% (group 2). Nursing staff care was rated positively by 100% of patients in both groups, with a predominance of very good ratings, especially in the operative cohort (87.2%).

Interdisciplinary care provided by other departments (e.g., Nuclear medicine, Otorhinolaryngology, Anesthesiology, Internal medicine) was also rated favorably, with very good or good ratings in 88.9% (diagnostic group) and 92.3% (group 2).

Adequate information regarding pre-treatment and preoperative diagnostic procedures was reported by most patients in both cohorts (95.6% diagnostic vs. 97.4% operative).

#### 3.3.1. Operative Procedures and Perioperative Patient Information

Surgery was performed in 75.6% of patients of diagnostic cohort, predominantly biopsies (71.1%). In contrast, the operative cohort underwent tumor resections, most frequently at the floor of the mouth (FOM) and tongue (51.3%), maxillary (17.9%) and mandibular resections (12.8%). Within the operative cohort, neck dissection (ND) was performed in 53.8% of cases and tracheostomy was required in 46.2%. Microvascular free-flap reconstruction was conducted in 25.6% of patients, including 30% with free vascularized bone flaps.

Patient information was rated positively in both cohorts. Adequate information provided during preoperative informed consent was rated as very good or good by 97.1% of patients in the diagnostic cohort and 97.4% in the operative cohort, with a higher proportion of very good ratings observed in the operative cohort (61.5% vs. 47.1%). Furthermore, 92.3% of operative patients reported feeling sufficiently informed about the postoperative course (e.g., tracheostoma, restrictions in speech and oral intake, drain removal, pain, suture or staple removal) as well as anticipated functional limitations. Reported information gaps were rare and mainly concerned isolated aspects such as tracheostoma care, postoperative pain or individual concerns.

#### 3.3.2. Discharge Management

Discharge organization was generally rated positively, with most patients reporting good or very good experiences. In the diagnostic cohort, 86.4% rated the discharge organization as very good or good, whereas 6.8% perceived it as less good and 6.8% reported uncertainty. Similarly, in the operative cohort, 75% rated the discharge organization as very good or good, while 13.9% rated it as less good and 11.1% were unsure.

Most patients reported having been informed about their upcoming discharge in a timely manner. This applied to 97.8% of patients in the diagnostic cohort and 92.3% in the operative cohort.

Regarding the timing of discharge notification, patients in both cohorts were most frequently informed one day prior to discharge (group 1: 46.7%; group 2: 28.2%). A relevant proportion of operative patients reported being informed two or more days in advance, whereas same-day notification was more common in the diagnostic cohort.

Most respondents felt adequately prepared for discharge. In the diagnostic cohort, 92.9% rated their discharge preparation as very good or good, compared with 82.1% in the operative cohort.

An offer of further or follow-up treatment was reported by 58.1% (group 1) and 62.2% (group 2). When follow-up care was desired, it was predominantly organized by the hospital (group 1: 95.7%; group 2: 85.7%). Discrepancies between the number of follow-ups offers and affirmative responses reflect cases in which patients declined further care or did not complete the corresponding questionnaire items.

Notably, perceptions of the hospital discharge management itself differed markedly between cohorts. While 75% of diagnostic patients rated discharge management as very good or good, 40.7% of operative patients provided a positive rating, with a substantial proportion indicating uncertainty. Utilization of hospital’s discharge management service was reported by 30.3% of diagnostic and 18.2% of operative patients. The high rate of missing or uncertain responses suggests limited patient awareness of the service or questionnaire completion prior to discharge. Although operative patients tended to report greater uncertainty regarding discharge management, no statistically significant differences between cohorts were observed after confirmation using Fisher’s exact test (*p* = 0.0559).

Significant sex-specific difference was identified in the evaluation of discharge management offers (*p* = 0.0112). These sex-stratified analyses were exploratory, based on small subgroup sizes and should therefore be interpreted with caution. Females more frequently selected “don’t know” (*n* = 15), whereas male patients more often rated the offer as good (*n* = 16) and very good (*n* = 9). No other discharge-related item demonstrated significant sex-based differences. Apart from this, no consistent sex-specific differences were observed across the remaining assessed domains.

### 3.4. Supportive Care Services

Across supportive care domains, utilization patterns varied by treatment intensity, with speech therapy showing a strong association with surgical extent (*p* < 0.001), whereas psycho-oncological and nutritional counseling were accessed less frequently but received consistently high ratings. Overall, more than 95% of respondents evaluated supportive care as good or very good.

#### 3.4.1. Psycho-Oncology

Psycho-oncological needs screening was reported by only a minority of patients in both cohorts (group 1: 26.7%; group 2: 23.1%). In contrast, an offer of psycho-oncological counseling was reported more frequently, particularly in the operative cohort (group 1: 46.7%; group 2: 61.5%). Among patients who received such an offer, 23.8% in group 1 and 25% in group 2 reported having utilized psycho-oncological counseling.

Counseling was rated as very good or good by 80% of respondents in the diagnostic cohort and by 83.3% in the operative cohort, with one negative evaluation reported in group 2. Most users reported a perceived benefit from psycho-oncological counseling. In group 1, 60% stated that they had benefited, whereas 20% reported no benefit and 20% were unsure. In group 2, 83.3% of patients reported a perceived benefit, with the remaining respondent indicating uncertainty. No statistically significant differences between cohorts were observed.

#### 3.4.2. Social Services

An offer of hospital social service was made to 26.7% of patients in the diagnostic cohort and to 48.7% of patients in the operative cohort, indicating significantly higher availability among operative patients (Fisher’s exact test, *p* = 0.031). In patients undergoing major surgical procedures, social service counseling was offered significantly more frequently (Fisher’s exact test, *p* = 0.042). Accordingly, utilization of social service counseling was significantly higher in this cohort (Fisher’s exact test, *p* = 0.027). All diagnostic patients who utilized this service rated it as very good [90.9%: very good or good (group 2)]. A perceived benefit was reported by all diagnostic patients and by 54.5% of operative patients, where uncertainty regarding its benefit was more frequently.

#### 3.4.3. Nutritional Counseling

The offer of nutritional counseling was reported by a minority of patients in both cohorts (group 1: 13.3%; group 2: 12.8%). Among patients who received this offer, utilization remained limited, with 33.3% in the diagnostic cohort and 40% in the operative cohort making use of the service. Notably, all patients who utilized nutritional counseling reported a perceived benefit from the consultation.

#### 3.4.4. Speech Therapy

This supportive care domain was reported by a small proportion of the diagnostic cohort (6.7%). In contrast, speech therapy was reported by 46.2% of operative patients. In the diagnostic cohort, all treated patients (100%) rated this therapy as very good or good. Similarly, in the operative cohort, patient-reported experience was predominantly positive, with 88.9% rating as good or very good. A perceived benefit from speech therapy was reported by all treated patients (100%) in the diagnostic cohort, whereas in the operative cohort, 72.2% indicated that they had benefited. Overall, speech therapy was significantly more frequently applied in the operative cohort (Fisher’s exact test, *p* < 0.001).

### 3.5. Influence of Surgical Extent

Patients undergoing more extensive surgical procedures (defined by presence of tracheostoma, neck dissection or reconstructive surgery) did not differ in their perceived adequacy of surgical information compared to patients with less extensive procedures. However, patients with extensive surgery were informed significantly earlier about their planned discharge date and were more frequently offered social service counseling and logopedic therapy.

### 3.6. Factors of Overall Satisfaction

Overall satisfaction with oncologic care was high, with >90% of patients rating their overall treatment experience as good or very good. Only 3 patients reported an overall negative experience.

Statistical analysis revealed that dissatisfaction was strongly associated with insufficient information regarding diagnostic procedures and tumor-related counseling. Both factors were significantly associated with overall treatment satisfaction (Fisher’s exact test, *p* < 0.001 for both). In contrast, time to surgery was not associated with overall satisfaction.

Qualitative analysis of free-text responses identified long waiting times as the most frequently reported source of criticism (*n* = 13), predominantly related to surgical treatment (*n* = 8) and diagnostic procedures (*n* = 5). Additional concerns included perceived staff shortages or limited staff availability (*n* = 4) as well as difficulties with orientation within the hospital facilities (*n* = 3).

Exploratory sex-stratified analyses were performed across selected questionnaire domains. A sex-specific difference was observed only for the evaluation of discharge management offers, with female patients more frequently selecting “don’t know”, whereas male patients more often provided positive ratings. No consistent sex-related differences were identified in the remaining domains. Given the small subgroup sizes, these analyses were underpowered and should be interpreted cautiously.

## 4. Discussion

This prospective cross-sectional study provides an exploratory evaluation of patient satisfaction and supportive care pathways within a certified German HNCC embedded in an university-based Comprehensive Cancer Center (CCC).

Overall, patient-reported satisfaction across diagnostic and surgical care pathways was consistently high, emphasizing the effectiveness of structured interdisciplinary and multiprofessional management in head and neck oncology [48]. Within the limitations of this exploratory design, the findings indicate that discharge coordination, adequacy of information and the integration of supportive care disciplines are closely associated with perceived overall care quality [49,50,51,52].

### 4.1. Patient Satisfaction Across Diagnostic and Surgical Pathways

A central observation of this study is that no statistically significant differences in patient satisfaction were detected between diagnostic and operative cohorts across most evaluated domains, including admission procedures, inpatient organization, medical and nursing care, and perioperative information. However, given the limited sample size, the absence of statistically significant differences does not exclude the possibility of true underlying differences that could not be detected due to insufficient statistical power. This aligns with previous reports suggesting that structured multidisciplinary care models may be associated with a high level of perceived care quality across varying levels of clinical complexity [53,54].

In head and neck oncology, where diagnostic work-up and surgical treatment are often associated with anxiety, uncertainty and functional impairment, consistent communication and continuity of care represent relevant components of patient experience [55]. The uniformly high ratings observed in our cohort reinforce the importance of standardized communication pathways and repeated patient counseling embedded within certified tumor center structures [48,56].

### 4.2. Discharge Management as a Critical Interface of Care

Discharge management emerged as a particularly sensitive domain. Although overall ratings were positive, operative patients showed a higher degree of uncertainty and lower utilization of formal discharge management services compared with diagnostic patients [57]. While this difference did not reach statistical significance, the observed distribution may indicate a potential difference that warrants further investigation in larger cohorts. However, this finding should be interpreted cautiously and cannot be considered confirmatory. Given the exploratory nature of this study, these findings should be interpreted cautiously. They may indicate that hospital discharge represents a vulnerable transition point, particularly in surgically treated HNC patients who frequently face functional limitations, complex aftercare requirements and psychosocial stressors [58,59].

Importantly, all sex-stratified analyses were exploratory and underpowered because of the limited subgroup sizes and should therefore be interpreted with caution and not as confirmatory findings. These findings may reflect differences in information needs, communication preferences or expectations regarding care coordination [60,61]. However, such findings also suggest that most observed sex differences in patient-reported outcomes, including aspects of health-related quality of life, likely reflect sex-related differences that are also observed in the general population and therefore may not be cancer-specific, as supported by previous literature [62]. It is possible that women place greater emphasis on anticipatory guidance and detailed discharge planning; however, this interpretation remains speculative. Targeted optimization of discharge counseling, including explicit explanation of available supportive care services and earlier involvement of discharge coordinators or care navigators, has been shown to improve patient experience and readiness for discharge and may therefore enhance perceived care quality during this vulnerable transition phase [63,64,65].

### 4.3. Role and Utilization of Supportive Care Services

Supportive care services were consistently rated positively by patients who accessed them. However, utilization rates varied across services and patient groups. Operative patients more frequently received and utilized social work counseling and speech therapy, particularly in the context of extensive surgical procedures. This pattern may reflect appropriate needs-based allocation of supportive care resources, as patients undergoing extensive interventions typically face greater functional and socioeconomic challenges [2,66,67]. However, due to the exploratory nature of the analysis, these associations should not be interpreted as causal.

In contrast, psycho-oncological and nutritional counseling were utilized less frequently despite being offered at comparable rates. This observation is consistent with previous reports of underutilization of psycho-oncological support in HNC populations [68,69]. Potential explanations include limited patient awareness, stigma or timing of assessment [70,71]. In selected cases, watchful-waiting may be appropriate, as nearly one third of HNC patients with psychological symptoms may recover spontaneously [72]. These observations suggest the importance of proactive screening and the early, repeated offering of supportive services throughout treatment and aftercare [73]. Nevertheless, these interpretations remain speculative and require confirmation in prospective studies.

### 4.4. Factors of Overall Satisfaction

An association was observed between overall satisfaction and the perceived adequacy of information regarding diagnostic procedures and tumor-related counseling. Patients reporting dissatisfaction more frequently indicated deficiencies in these domains, whereas objective factors such as waiting time to surgery were not associated with negative overall ratings [74,75]. These findings suggest that patient experience may be more closely related to perceived communication quality than to objective process parameters [22,76]. This aligns with established quality-of-care frameworks, in which communication and information transfer constitute central process indicators [77,78].

### 4.5. Clinical Implications

Within the limitations of this exploratory analysis, several implications may be considered. Structured interdisciplinary care pathways within certified tumor centers appear to be associated with high levels of patient-reported satisfaction. Discharge management may represent a relevant area for further optimization, particularly in surgically treated patients. Additionally, proactive integration of supportive care services based on structured screening may improve patient access to these resources. These considerations should be interpreted as hypothesis-generating and require validation in larger, preferably multicenter studies.

### 4.6. Limitations

Several limitations must be considered when interpreting these findings. First, this study was conducted as a single-center investigation within a certified, highly specialized HNCC, which may limit generalizability to non-certified or lower-volume institutions. Second, although data were collected prospectively, the cross-sectional design captures patient experience at a single time point and precludes any assessment of longitudinal changes or causal relationships. Accordingly, all observed associations must be interpreted as non-causal. Additionally, due to the fully anonymous study design, no data on non-participants or individuals who declined participation were recorded, which may introduce selection bias and limit the assessment of potential differences between respondents and non-respondents.

Third, the study was designed as an exploratory analysis without formal sample size calculation. The overall sample size and particularly the low utilization rates of certain supportive care services resulted in small subgroup sizes, thereby limiting statistical power and precluding confirmatory subgroup analyses. This particularly applies to sex-stratified subgroup analyses, which were exploratory and underpowered and therefore do not permit robust conclusions regarding sex-specific differences. Consequently, statistical findings should be considered descriptive and hypothesis-generating and must be interpreted with caution.

Fourth, the questionnaire used in this study was institution-specific and was not formally validated with respect to psychometric properties. No formal assessment of reliability or construct validity was performed. The absence of standardized validation procedures may introduce measurement bias and limit the comparability and generalizability of the findings.

Fifth, no correction for multiple testing was applied, increasing the risk of type I error. Therefore, reported *p*-values should be interpreted as descriptive indicators rather than confirmatory evidence.

Finally, although questionnaires were completed anonymously, response bias cannot be fully excluded, particularly considering the very high satisfaction rates observed.

Social desirability bias may have contributed to systematically more favorable responses. In combination with the generally high satisfaction ratings observed in this cohort, this suggests the presence of ceiling effects, which may limit the discriminatory capacity of the instrument and potentially inflate positive evaluations.

### 4.7. Future Directions

Future studies should focus on longitudinal assessment of patient satisfaction and patient-reported outcomes across the full oncologic care continuum, including outpatient follow-up and survivorship. Integration of digital Patient-Reported Outcome Measures (PROM) platforms or applications and real-time feedback systems may further enhance responsiveness of care pathways. Multicenter studies are warranted to establish benchmarks for patient satisfaction and supportive care integration in head and neck oncology at a national and international level.

## 5. Conclusions

This study describes consistently high patient satisfaction within a certified German HNCC. Adequate and transparent patient information regarding diagnostic procedures and tumor-related counseling was frequently reported in association with higher overall satisfaction, whereas objective factors such as waiting time were not associated with negative ratings. Supportive care services were generally perceived positively by patients, although utilization varied across domains. While utilization of supportive disciplines remained limited, satisfaction among patients was uniformly high, highlighting the importance of proactive, needs-based integration. Discharge management was identified as a potentially sensitive transition point, particularly in surgically treated patients. Given the exploratory design, limited sample size and cross-sectional nature of this study, all findings should be interpreted with caution and considered hypothesis-generating. Further studies with larger cohorts and longitudinal designs are required to validate these observations and to better define determinants of patient satisfaction in oncology care.

## Figures and Tables

**Table 1:** Sex distribution of the study cohort stratified by study group. Data are presented as absolute numbers and percentages (*n*, %). Percentages were calculated within each study group. The diagnostic cohort includes patients undergoing tumor staging, whereas the operative cohort comprises patients undergoing surgical treatment. “Not reported” indicates missing information on sex.

Sex	Diagnostic Cohort (*n* = 45)	Operative Cohort (*n* = 39)	Total (*n* = 84)
Female	15 (33.3)	19 (48.7)	34 (40.5)
Male	27 (60.0)	18 (46.2)	45 (53.6)
Not reported	3 (6.7)	2 (5.1)	5 (6.0)

## Data Availability

The original contributions presented in this study are included in the article/Supplementary Material. Further inquiries can be directed to the corresponding author.

## Supplementary Materials

The following supporting information can be downloaded at: https://www.mdpi.com/article/10.3390/healthcare14091192/s1, Table S1: STROBE checklist for reporting of this prospective cross-sectional study.

## Abbreviations

The following abbreviations are used in this manuscript:

CCC	Comprehensive Cancer Center
DKG	German Cancer Society (or Deutsche Krebsgesellschaft)
DT	Distress Thermometer
ECOG	Eastern Cooperative Oncology Group
ESPEN	European Society for Clinical Nutrition and Metabolism
[^18^F] FDG PET/CT	Fluorine-18 fluorodeoxyglucose positron emission tomography combined with computed tomography
FOM	Floor of the mouth
HNC	Head and neck cancer
HNCC	Head and Neck Cancer Center
HNSCC	Head and neck squamous cell carcinoma
HNTB	Head and neck tumor board
HRQoL	Health-Related Quality of Life
ICD-10	International Statistical Classification of Diseases and Related Health Problems (10. revision)
KPS	Karnofsky performance status
MAR	Missing-at-random
MRI	Magnetic resonance imaging
NCCN	National Comprehensive Cancer Network
ND	Neck dissection
NRS	Nutritional Risk Screening
OMF	Oral and maxillofacial
POD	Postoperative day
PROM	Patient-Reported Outcome Measures
QOL	Quality of life
STROBE	Strengthening the Reporting of Observational Studies in Epidemiology
