# Supplementary material for: Patient Satisfaction and Supportive Care Pathways in a German Head and Neck Tumor Center: A Prospective Cross-Sectional Study
Source: Healthcare (Basel). 2026 Apr 29;14(9):1192. doi: 10.3390/healthcare14091192 (PMC13163697; doi:10.3390/healthcare14091192)
Supplement: Supplementary file 1 [file healthcare-14-01192-s001.zip › healthcare-4192407-supplementary.pdf]

healthcare MDPI

STROBE Checklist—Cross-Sectional Study

Supplementary Table S1. STROBE checklist for reporting of this prospective cross-sectional study. This table summarizes the adherence of the present study to the Strengthening the Reporting of Observational Studies in Epidemiology (STROBE) guidelines for cross-sectional studies. STROBE items are listed with the corresponding implementation in the manuscript.

Item	Recommendation	Reported in Manuscript
1(a)	Study design in title/abstract	Cross-sectional study stated in title
1(b)	Informative abstract	Structured abstract provided
2	Background/rationale	Epidemiological and clinical context described
3	Objectives	Clearly defined study aim
4	Study design	Prospective cross-sectional design stated
5	Setting	Ulm, Head and Neck Cancer Center (HNCC), March 2023 - April 2025
6(a)	Eligibility criteria	Defined inclusion/exclusion criteria
6(c)	Non-participants	Not recorded due to anonymity
7	Variables	Patient satisfaction, supportive care defined
8	Data sources	Standardized questionnaire used
9	Bias	Selection, response, measurement bias discussed
10	Study size	Exploratory, no formal calculation
11	Quantitative variables	Ordinal Likert-scale data
12(a)	Statistical methods	Pearson's chi-square, Fisher`s tests
12(c)	Missing data	Case-wise exclusion
12(d)	Subgroup analyses	Exploratory, underpowered
13(a)	Participants	84 included, exclusions reported
14(a)	Descriptive data	Cohort characteristics provided
15	Outcome data	Satisfaction outcomes reported
16(a)	Main results	Descriptive statistics, p-values
19	Limitations	Comprehensive limitations section
20	Interpretation	Cautious, hypothesis-generating
21	Generalizability	Limited generalisability discussed
22	Funding	No external funding
